# Thermochromic phantom for therapeutic ultrasound daily quality assurance

**DOI:** 10.1186/2050-5736-3-S1-P72

**Published:** 2015-06-30

**Authors:** Farhan Qureshi, Zachary Larrabee, Chris Roth, Arik Hananel, Matt Eames, David Moore, John Snell, Neal Kassell, Jean-Francois Aubry

**Affiliations:** 1 Focused Ultrasound Foundation, Charlottesville, Virginia, United States; 2 Institut Langevin, Paris, France

## Background/introduction

Errors in power output ranging from −100% to +210% have been reported in a multitude of physiotherapy transducers.[1] Differences in power output can arise even after careful calibration on an annual or bi-annual schedule, which can either result in harm to the patient or non-effective treatment. Therefore, easy implementation of daily quality assurance is of great importance. We propose a simple, easy to use DQA phantom which allows the user to assess the power output of the focused ultrasound transducer, and determine if it has changed significantly after calibration. The basis of this phantom is the use of a highly attenuating ultrasound absorber with a surface layer of thermochromic liquid crystals (TLC). The use of thermochromic materials as a technique for use in ultrasound phantoms has been an ongoing area of interest. Many of these techniques require complicated set-ups, with large water tanks and imaging systems to produce very accurate data.[2,3] Simpler phantoms have been developed for use in benchtop settings, but still with a large emphasis on complicated image analysis.[4] Our proposed phantom will be a product where the end-user can visually assess the size of the lesion formed as a function of power output, without resorting to complicated image analysis.

## Methods

In the phantom, differences in lesion size due to differences in ambient temperature are revealed by visual inspection. A simple temperature scale will correspond to concentric circles of varying radii printed on the phantom. The generated lesion size matches the radius of the corresponding circle at each ambient temperature, giving a visual check as to whether or not the transducer’s power output has changed. A Matlab code has been developed to numerically solve the bio-heat equation in finite differences time domain with a generation term dependent on the induced pressure field. The code generates plots with a color map corresponding to the thermochromic material’s color changing properties, and the acoustic absorber’s acoustic attenuation and thermal diffusivity properties. The attenuation coefficient of the material, provided by Precision Acoustics Ltd, was 28.8 ±2.6 dB∙cm-1∙MHz-1. The thermal diffusivity was tested by Decagon Devices, Inc. using the transient line heat source method, and was determined to be 0.09 ±0.01 mm2/s. The peak positive pressure is adjusted in the simulation so that the lesion size generated by the simulation corresponds to the actual lesion size measured after sonication, giving an estimate of the pressure at focus.

## Results and conclusions

A method for creating a simple DQA phantom has been devised. The main goal of the phantom is to verify that the output power of the transducer does not change compared to initial calibration. The end user places the phantom in their transducer system, and sonicates for a set power and time. The power output of the transducer is then checked visually using a system of concentric circles printed on the phantom. This system is a cheap and effective way to produce DQA phantoms, both because of the ease of use, and because the use of TLC makes visual changes in the phantom reversible and reproducible over a long period of time.

**Figure 1:**
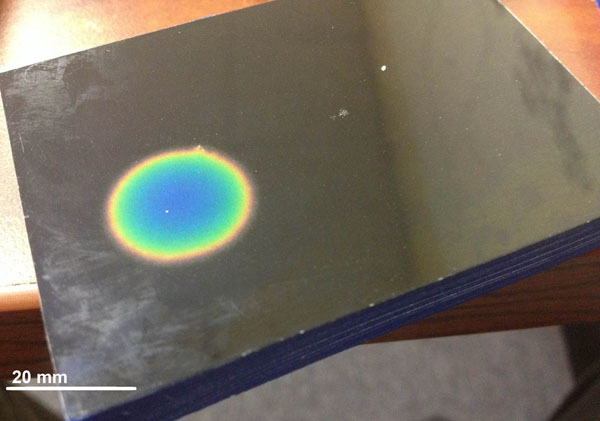
Phantom after Sonication with HIFU Transducer

**Figure 2:**
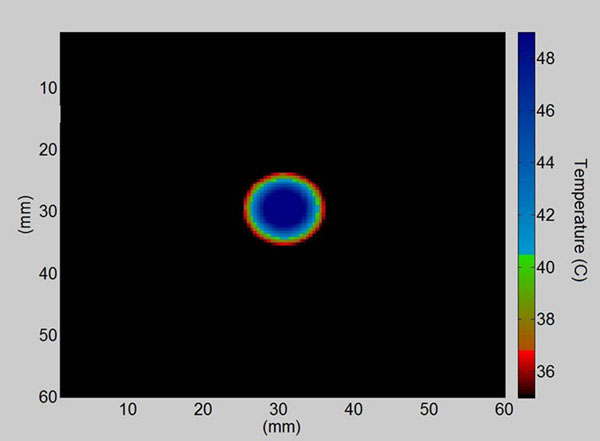
Simulated Temperature Elevation
